# An International Multicentre Retrospective Cohort Study Evaluating Robot-Assisted Total Mesorectal Excision in Experienced Dutch, French, and United Kingdom Centres—The EUREKA Collaborative

**DOI:** 10.3390/cancers17081268

**Published:** 2025-04-09

**Authors:** Ritch T. J. Geitenbeek, Charlotte M. S. Genders, Christophe Taoum, Rauand Duhoky, Thijs A. Burghgraef, Christina A. Fleming, Eddy Cotte, Anne Dubois, Eric Rullier, Quentin Denost, Jim S. Khan, Roel Hompes, Philippe Rouanet, Esther C. J. Consten

**Affiliations:** 1 Department of Surgery, University Medical Center Groningen, University of Groningen, 9713 GZ Groningen, The Netherlandscms.genders@meandermc.nl (C.M.S.G.);; 2 Department of Surgery, Meander Medical Center, 3813 TZ Amersfoort, The Netherlands; 3 Surgery Department, Montpellier Cancer Institute (ICM), University of Montpellier, 34090 Montpellier, France; 4 Department of Colorectal Surgery, Portsmouth Hospitals University NHS Trust, Portsmouth PO6 3LY, UK; 5 Bordeaux Colorectal Institute, Clinique Tivoli, 33300 Bordeaux, France; 6 Department of Digestive and Oncological Surgery, Lyon University Hospital, Lyon-Sud Hospital, 69495 Pierre-Bénite, France; 7 Department of Colorectal Surgery, Chu Estaing, 63100 Clermont-Ferrand, France; 8 Colorectal Unit, Department of Digestive Surgery, Haut-Lévêque Hospital, Bordeaux University Hospital, 33600 Pessac, France; 9 Department of Surgery, University Medical Center Amsterdam, University of Amsterdam, 1105 AZ Amsterdam, The Netherlands; 10 Department of Surgery, Amsterdam Cancer Center, 1081 HV Amsterdam, The Netherlands

**Keywords:** international multicentre cohort, robot-assisted surgery, rectal cancer, total mesorectal excision

## Abstract

Background: Robot-assisted total mesorectal excision has been proposed as an alternative to laparoscopic TME for rectal cancer. However, its short-term outcomes and long-term oncological efficacy remain debated, especially in Western populations. This study evaluates the short-term clinical and long-term oncological outcomes of robot-assisted total mesorectal excision performed by experienced surgeons in high-volume European centres. Methods: This multicentre, international, retrospective cohort study included 1390 patients from the EUREKA collaborative dataset who underwent robot-assisted total mesorectal excision for rectal cancer between January 2013 and January 2022. All surgeries were performed by expert surgeons beyond the learning curve. Data were analysed for patient demographics, perioperative outcomes, pathological findings, and three-year survival metrics. Kaplan–Meier analysis was used to evaluate overall and disease-free survival. Results: Of 1390 patients, 60.6% underwent restorative low anterior resection. Conversion to open surgery occurred in 3.7%, and postoperative complications were reported in 28.7%. Anastomotic leakage occurred in 14.7% of patients who underwent restorative low anterior resection. The median operative time was 223 min. R0 resection was achieved in 94.7%, and circumferential resection margin positivity was 5.5%. Three-year overall survival was 90.1%, disease-free survival was 88.6%, and local recurrence was 2.9%. Conclusions: Robot-assisted total mesorectal excision performed by experienced surgeons in high-volume European centres is safe, with low conversion rates, acceptable complication rates, and favourable oncological outcomes. These findings underscore the potential of robot-assisted total mesorectal excision as a standard approach for rectal cancer in specialised settings.

## Simple Summary

This international, retrospective cohort study presents the results of robot-assisted total mesorectal excision performed in high-volume rectal cancer centres. The results demonstrate that robot-assisted surgery offers favourable outcomes, with promising short-term results and oncological safety.

## 1. Introduction

Laparoscopic total mesorectal excision (L-TME) has been widely adopted due to its superior postoperative outcomes compared to open surgery [1,2]. However, despite extensive randomised controlled trials (RCTs) [3,4], the oncological efficacy of L-TME has not been definitively established over traditional open surgery. The primary limitations of the laparoscopic technique, including suboptimal visibility and restricted access within the low pelvis, pose challenges in dissection and distal division, which may lead to incomplete resections [4]. These technical constraints have prompted the exploration of alternative surgical techniques.

Robot-assisted total mesorectal excision (R-TME) has emerged as a promising innovation in rectal cancer surgery [5], offering enhanced 3D visualisation, improved instrument precision and a stable camera platform, which may overcome the limitations of conventional laparoscopy. Nevertheless, early RCTs [6,7] failed to demonstrate significant benefits of the robot-assisted technique compared to laparoscopy, aside from a lower conversion rate in obese male patients. Similarly, the COLRAR trial [6], which was prematurely halted, did not report significant differences in pathological or postoperative outcomes between the two techniques. However, these early studies were conducted during the initial phase of robot-assisted surgery adoption, when surgeon experience was still limited [8].

As robot-assisted surgery expertise has grown and high-volume centres have become more prevalent, more recent trials, such as the REAL trial [9], suggest that R-TME may offer improved resection quality, reduced operative trauma, and enhanced recovery. However, the REAL trial [9] was conducted in high-volume centres in Asia, with different patient demographics, limiting the applicability to Western demographics.

Furthermore, systematic reviews and meta-analyses have indicated potential advantages of R-TME over L-TME in areas such as conversion rates, circumferential resection margin (CRM) involvement, length of stay, and complication rates [10,11,12]. Nevertheless, the most recent meta-analysis by Simillis et al. [2] included studies until September 2018, with a substantial focus on East Asian populations, which may not accurately reflect outcomes in Western populations. There remains a critical need for robust, real-world data on the comparative effectiveness of laparoscopic and robot-assisted TME beyond the learning curve, particularly in European healthcare systems.

To address this knowledge gap, we conducted an international, multicentre, retrospective study, drawing on data from expert surgeons beyond the learning curve in leading centres across the Netherlands, France, and the United Kingdom. This study aims to provide comprehensive insights into the real-world outcomes of R-TME, thereby advancing our understanding of its clinical benefits and limitations in Western populations.

## 2. Materials and Methods

### 2.1. Study Design

This international, retrospective, multicentre cohort study reported the outcomes of R-TME for rectal tumours performed from January 2013 to January 2022. The study utilised data from an established dataset known as the Expert Dutch, French, and UK Robotic Rectal Cancer Centres (EUREKA) collaborative dataset. A protocol regarding the design, methods, and data points of the EUREKA collaborative dataset has been previously published [13]. Data were sourced from tertiary referral centres specialised in colorectal cancer across the Netherlands, France, and the United Kingdom. All centres maintained prospective research databases. Surgical procedures were performed by expert robot-assisted surgeons, defined as those who had surpassed their learning curve for robotic rectal surgery. This threshold was set at ≥35 procedures, based on evidence from Burghgraef et al., who assessed the learning curve for robotic TME and demonstrated that proficiency was typically achieved between 12 and 35 cases [14]. We applied the upper limit (35 cases) as the cutoff to define an “expert surgeon” in this study. All cases were reviewed in multidisciplinary team meetings and treated according to local and national guidelines. Ethical approval was secured from all participating centres’ institutional review boards in compliance with international research standards. This study was conducted following the “strengthening the reporting of observational studies in epidemiology guidelines for observational studies” (STROBE) guidelines [15] (Supplementary File S1).

### 2.2. Patients

Included were patients (1) aged ≥ 18 years; (2) diagnosed with a rectal tumour located within ten centimetres from the anorectal junction (ARJ) on magnetic resonance imaging (MRI); and (3) who underwent a curative robot-assisted proctectomy with partial or complete TME between January 2013 and January 2022. Exclusion criteria were (1) patients undergoing rectal cancer surgery as part of an extended procedure and (2) patients who had palliative or emergency resections. Complete responders to neoadjuvant chemoradiotherapy (nCRT) were not excluded, allowing for a comprehensive evaluation of the entire patient cohort. We accounted for missing data of up to 30% in outcome variables, recognising the variability in national practices and the study’s retrospective nature.

Preoperative diagnostics included patient demographics, tumour histology, and cancer staging, including chest, abdomen, and pelvis computed tomography (CT) scan and pelvic MRI. Various neoadjuvant and adjuvant therapies were utilised depending on patient and tumour characteristics. Commonly used neoadjuvant therapies included nCRT, short-course radiotherapy (SCRT), total neoadjuvant therapy (TNT) (addition of induction or consolidation with nCRT or SCRT) generally using FOLFOX or FOLFIRINOX. Some local differences in practice between participating centres should be noted. TNT is quite frequently practised in French rectal cancer centres, with the use of FOLFIRINOX as the preferred systemic chemotherapy agent [16]. There is also a culture of sphincter preservation even for tumours < 5 cm from the anal verge with transanal low rectal dissection commonly performed to facilitate this [17]. Finally, adjuvant chemotherapy is frequently administered for ypN+ disease and is supported by French national guidelines [18]. Neoadjuvant chemo- and radiotherapy are less commonly used in the United Kingdom than in other participating centres. Patients underwent short-course or long-course nCRT after multidisciplinary team discussion, with clinical restaging and surgery performed afterwards. nCRT regimens included either SCRT (25 Gy in 5 fractions) or long-course nCRT (45–50 Gy in 25 fractions) with concurrent chemotherapy (CAPOX or FOLFOX). Clinical and oncological outcomes were recorded in patient notes.

### 2.3. Surgical Procedure

The surgical approach for each patient was determined by a combination of surgeon preference, patient input, and available robotic platforms. All robot-assisted procedures were conducted using the da Vinci Si/X/Xi systems. Patients underwent mechanical and antibiotic bowel preparation prior to surgery [19], as per established protocols. Surgical resections adhered to standardised protocols for TME or partial mesorectal excision (PME, also referred to as tumour-specific mesorectal excision) [20,21], in accordance with national guidelines. For lower rectal tumours, TME was performed, involving the complete removal of the mesorectum down to the pelvic floor, applicable in both sphincter-preserving surgeries and abdominoperineal resections (APR). PME was conducted for upper rectal tumours, with resection of the mesorectum extending at least 5 cm distal to the lower tumour margin. Lymph node dissection at the origin of the inferior mesenteric artery was routinely performed, with hypogastric nerve preservation attempted when technically feasible. Conversion to open surgery and the decision to create diverting stomas were left to the operating surgeon’s discretion. APR was considered when sphincter preservation was not achievable. In cases requiring APR, the robotic platform was utilised for the abdominal phase, while the perineal phase was completed manually. All patients followed enhanced recovery after surgery (ERAS) protocols postoperatively, with detailed documentation of clinical and oncological outcomes. Postoperative management conformed to national follow-up protocols and standardised recovery guidelines. Consequently, the follow-up performed differed slightly per country, though generally involved follow-up visits along with laboratory and radiological evaluation through rectoscopy, MRI of the pelvis. Furthermore, CT scans with rectal contrast were performed prior to stoma reversal.

### 2.4. Outcome Parameters and Definitions

The primary aim of this study was to evaluate short-term clinical and long-term survival outcomes in patients undergoing R-TME within the EUREKA collaborative dataset [13]. The data points available in the EUREKA collaborative dataset [13] have been previously reported. In short, they consist of detailed retrospective data, including patient demographics, tumour staging, neoadjuvant treatment specifics, surgical details, postoperative clinical and histopathological outcomes, readmission rates, late morbidity, and long-term oncologic follow-up. The included variables are described in depth in Appendix A.

### 2.5. Statistical Analysis

All statistical analyses were performed using R version 4.3.023 (R Core Team, 2023) [22]. The percentages presented in the results represent the available data after excluding missing values. Categorical variables were reported as absolute case numbers and percentages, while continuous variables were summarised as mean values with corresponding standard deviations (SDs). Medians and interquartile ranges (IQRs) were presented for non-normally distributed continuous variables. Continuous variables, such as body mass index (BMI), were stratified into clinically relevant subgroups for analysis. Kaplan–Meier analysis of overall and disease-free survival was performed for all patients treated.

## 3. Results

**Figure 1:**
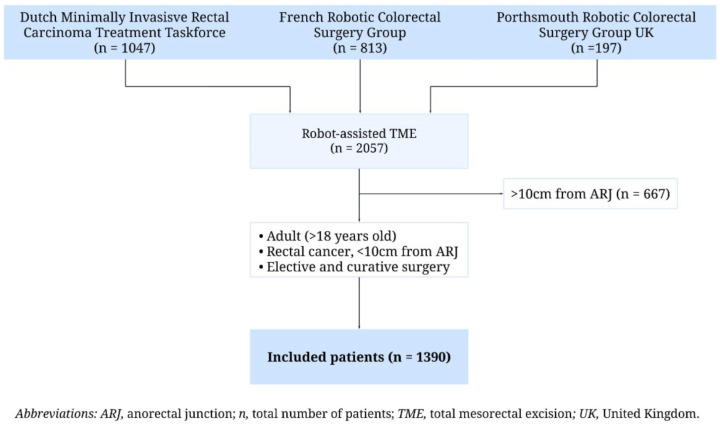
Flow diagram of included patients.

Between January 2013 and January 2022, 2057 patients who underwent R-TME were included in the EUREKA collaborative dataset [13]. After applying the inclusion and exclusion criteria, 1390 patients were eligible for analysis (Figure 1).

### 3.1. Baseline Characteristics

The cohort predominantly comprised male patients (65.3%), with the majority being overweight or obese (58.3%) (Table 1). At baseline, 8.6% of patients had clinical T4 (cT4) tumours, 53.8% had node-positive disease, and 7.4% had clinical evidence of distant metastasis. MRI revealed that mesorectal fascia (MRF) was involved in 44.1% of patients and extramural vascular invasion (EMVI) in 54.8%. The median tumour distance from the ARJ was 5 cm. A total of 830 patients (59.7%) received neoadjuvant treatment, with 283 (34.1%) undergoing nCRT and 281 (33.8%) receiving TNT. Among the patients who received neoadjuvant therapy for tumour downsizing, 323 (84.3%) exhibited a favourable tumour regression grade (TRG) response on MRI, while 60 (15.7%) showed no significant response.

### 3.2. Intraoperative Results

The majority of patients (60.6%) underwent a restorative low anterior resection (RLAR) (Table 2). Intraoperative complications were reported in 67 patients (5.5%), with conversion to open or laparoscopic surgery required in 52 cases (3.7%). Among the RLAR cohort, indocyanine green (ICG) fluorescence was used in 376 patients (45.7%) to assess perfusion before anastomosis formation, and 492 patients (58.4%) received a diverting stoma. The median total operative time was 223 min.

### 3.3. Postoperative Outcomes

The median length of postoperative hospital stay, excluding readmissions, was seven days. Within 31 days following surgery, 302 patients (28.7%) experienced postoperative complications (Table 3). Postoperative complication rates did not significantly differ between surgical procedures, with rates of 22.7% for RLAR, 19.3% for APR, and 21.1% for NRLAR. Readmission within 30 days occurred in 13.6% of patients, of which 78.2% required surgical reintervention. Over the entire follow-up period, 226 patients (16.3%) developed major complications requiring further surgical intervention (Clavien–Dindo grade ≥ 3). Anastomotic leakage was observed in 115 patients who underwent RLAR (14.7%), with early leakages being the most common.

### 3.4. Pathological Outcomes

Among the 1390 patients, 661 (47.8%) were found to have pT3-T4 tumours on final pathology, and 486 (35%) were pN+ (Table 4). The TME quality was deemed complete in 81.9% of specimens and near complete in 13.1%. CRM positivity was observed in 69 cases (5.5%), with rates stratified by tumour location: 3.7% in mid-rectal tumours (5–10 cm from the ARJ) and 5.9% in low rectal tumours (≤5 cm from the ARJ). R0 resection was achieved in 1180 patients (94.7%).

### 3.5. Long-Term Outcomes

**Figure 2:**
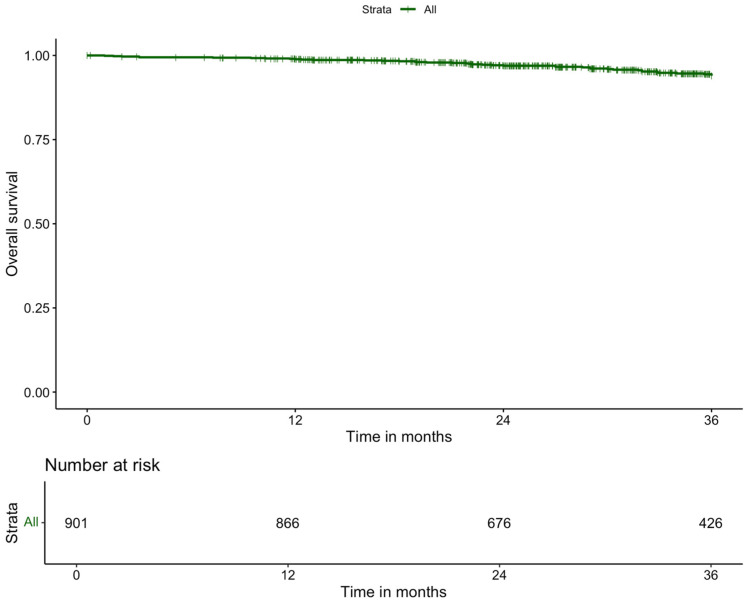
Kaplan–Meier curve of 3-year overall survival of patients with rectal cancer who underwent robot-assisted low anterior resection.

**Figure 3:**
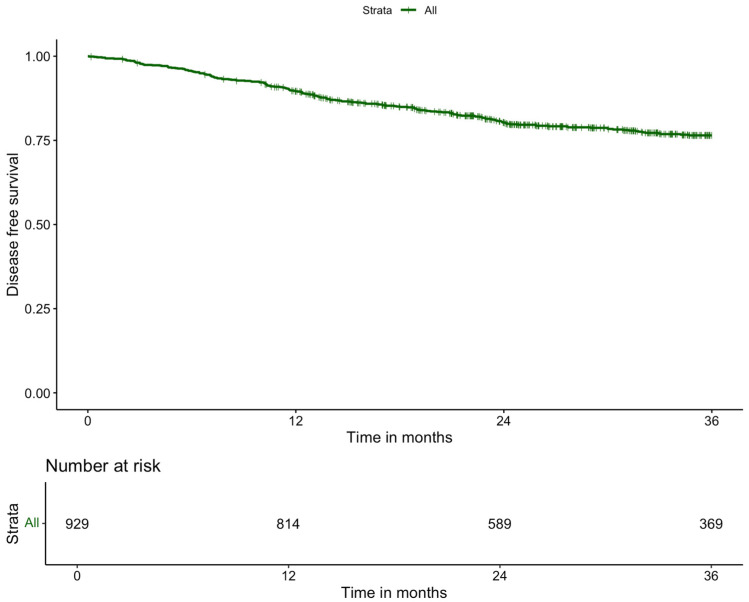
Kaplan–Meier curve of 3-year disease-free survival of patients with rectal cancer who underwent robot-assisted low anterior resection.

The median follow-up was 29.70 months (IQR: 18.46). Adjuvant chemotherapy was administered to 230 patients (17.7%) (Table 5). Local recurrence within three years occurred in 2.9% of patients, while the three-year systemic recurrence rate was 14.6%. The overall three-year survival rate was 90.1% (Figure 2), and the three-year disease-free survival rate was 88.6% (Figure 3).

## 4. Discussion

This large international, multicentre, retrospective cohort study evaluates the outcomes of R-TME for rectal cancer performed by experienced surgeons in high-volume tertiary centres. This study demonstrates that, in expert hands, robot-assisted surgery offers favourable outcomes in terms of conversion rates, postoperative complications, pathological outcomes, and three-year survival and recurrence rates.

The conversion rate to open surgery was 3.7%, reflecting the high-quality robot-assisted surgeries performed by expert surgeons. This rate is notably lower than the 8.1% conversion rate observed in the ROLARR trial [7], where surgeons were still within their learning curve. Although lower conversion rates have been reported in some Asian studies (e.g., COLRAR trial [6] (0.7%), and REAL trial [9] (1.7%)), these differences may be attributable to variations in patient demographics, such as BMI and tumour characteristics, suggesting that outcomes may differ across populations. Our conversion rate compares favourably to laparoscopic techniques, where conversion rates as high as 16% have been reported in RCTs such as the ROLARR trial [7], COLOR II trial [20], and RESET trial [23]. The low conversion rate in our cohort reinforces the technical precision afforded by robotic systems, especially in the narrow confines of the pelvis.

The operative time for R-TME in our cohort was a median of 223 min. In the early stages of adopting robotic techniques, it was suggested that the implementation of R-TME would lead to increased operative time and associated costs when compared to L-TME [24]. However, many of the early studies failed to account for the learning curve, which we know to be associated with surgical efficiency [14]. A lower operating time of 173 min was reported by the REAL trial [9]. Differences in the studied populations, regarding tumour stage and received neoadjuvant therapy, might pose more operative difficulties and explain a prolonged operative time. The operative time we found compares positively to the COLRAR trial [6] (265 min) and the ROLARR trial [7] (299 min), which did not account for the surgeon’s experience. This once more emphasises the importance of sufficient experience for surgeons performing R-TME. The operative time for R-TME in our cohort shows favourable comparison to Western L-TME cohorts reported in the ROLARR trial (261 min) [7] and COLLOR II trial (240 min) [20], demonstrating that R-TME can achieve operative efficiency equivalent to laparoscopic approaches when performed by experienced surgeons.

Postoperative complications occurred in 28.7% of patients, which aligns with the previously published systematic review and meta-analysis by Wang et al. [10]. Nevertheless, this rate is lower than the 33.1% complication rate reported in the ROLARR trial [7]. Notably, the ROLARR trial [7] did not account for the impact of the learning curve, in contrast to our study, which may have influenced the reported complication rates. While some studies, such as the REAL trial [9], report lower complication rates (16.2%), this trial excluded Clavien–Dindo grade I complications, limiting direct comparison. When viewed against laparoscopic cohorts, where complication rates of the ACOSOG Z6051 [3], ROLARR trial [7] and COLLOR II trial [20], range from 31.6% to 57.1%, our findings suggest that robot-assisted surgery might confer some advantages in reducing postoperative complications, potentially through enhanced precision and minimally invasive techniques.

In our cohort, anastomotic leakage was observed in 14.7% of patients who underwent RLAR. The majority of our patients underwent RLAR (60.6%). The anastomotic leakage rate is comparable to those reported in the RESET trial [23] (13.3%), and ROLARR trial [7] (12.2%). Conversely, some studies have reported lower leakage rates, underscoring the variability across the published literature. These discrepancies may be due to differences in leakage definition, detection methods, and follow-up duration. Our study utilised the International Study Group of Rectal Cancer (ISREC) classification [25] for anastomotic leakage, which provides a broad definition encompassing not only clinically significant leaks requiring intervention but also radiological leaks that may not have necessitated surgical management. This differs from studies such as the REAL trial [9], which primarily reported clinically significant leaks occurring in the early postoperative period. Furthermore, our study captured anastomotic leaks occurring throughout the full three-year follow-up period, whereas many trials reporting lower leakage rates focus on the immediate postoperative period, omitting leaks that develop beyond the initial hospitalisation phase. Moreover, our study included a high proportion of locally advanced rectal cancers, frequently located close to the ARJ and necessitating neoadjuvant therapy in the majority of cases, factors known to increase surgical complexity and the risk of anastomotic complications. Despite the relatively higher leakage rate in our study, our findings align with real-world data, such as the Dutch national audits [26], which a reported anastomotic leakage rate of 20%. The anastomotic leakage rate presented in our study is also similar to the Western L-TME cohorts from the ROLARR trial (12.2%) [7] and the COLLOR II trial (13%) [20]. Nonetheless, when comparing our results to these studies, caution is warranted due to the previously mentioned discrepancies in reporting of anastomotic leakages. This highlights the importance of standardising anastomotic leakage definitions and follow-up durations in future studies to improve comparability across different surgical approaches and populations.

A complete or nearly complete TME was achieved in 81.9% of patients. These results are better than the 75.4% completeness of TME rate reported in the ROLARR trial [7], which may be due to the current study only reporting outcomes of surgeons beyond the learning curve. Contrastingly, the REAL trial [9] reported a higher rate of 95.4% macroscopic completeness of resection rate after R-TME, which may be attributable to the previously mentioned differences in patient demographics. The RESET trial [23] reported an 89.7% complete TME, higher than the current study. That said, the RESET trial [23] excluded patients who underwent APR, which is known to be associated with higher rates of incomplete TME. Nevertheless, achieving an 81.9% rate of complete TME, despite a high prevalence of MRF positivity of 44.9%, highlights the utility of robotic platforms in facilitating meticulous dissection in anatomically challenging regions like the pelvis. Furthermore, these results are comparable to the complete and nearly complete TME rates in laparoscopic surgery, where RCTs [3,4,7,9,20] reported resection rates ranging from 75.2% to 91.8%.

The CRM positivity rate in this multicentre international study was 5.5%, aligning with previous robot-assisted trials [7,9] that reported CRM positivity rates ranging from 4.0% to 5.1%. In contrast, the RESET trial [23] reported a lower CRM positivity rate of robot-assisted sphincter-saving surgery of 1.8%. Despite the inclusion of advanced-stage tumours in the current study, the reported CRM positivity rate is lower compared to that of the laparoscopic arms of the ROLARR [7] (6.3%), ALaCaRT [4] (7%), REAL [9] (7.2%), and ACOSOG [3] (12.1%) trials. As having a positive CRM in the pathological specimen has been associated with local recurrence [27], the relatively low CRM positivity rate reported after robot-assisted surgery may lead to superior oncological safety compared to the laparoscopic technique. Nonetheless, any improvement in oncological outcomes after robot-assisted surgery leading to superior survival has yet to be demonstrated in RCTs with long-term follow-up.

In terms of oncological outcomes, our study demonstrated excellent three-year overall survival (90.1%) and disease-free survival (88.6%), with low local (2.9%) and systemic recurrence (14.6%) rates. Rectosigmoid cancers were excluded from the current study, as these tumours are associated with more favourable oncological and survival outcomes compared to rectal cancers [28]. This exclusion enhances the applicability of our findings to studies specifically evaluating rectal cancer. However, excluding rectosigmoid cancers may reduce the generalisability of the current study findings for prior studies including rectosigmoid cancers. The recurrence rates reported in our cohort are comparable to those reported by Sammour et al. [29]. Like the current study, this prospective single-centre cohort study included patients from a Western population with advanced-stage rectal cancer and demonstrated a local recurrence rate of 2.4% and a systemic recurrence rate of 16.9% [29]. The findings of the present study are favourable compared to laparoscopic trials like the COLOR II trial [30], which reported a three-year overall survival of 86.7% and a disease-free survival of 74.8%. Five-year survival and recurrence rates were unavailable in the current cohort, limiting the opportunity to compare our outcomes to studies reporting five-year survival, such as the study by Sammour et al. [29].

This study’s strengths include its large, multicentre design, which captures real-world practice in high-volume centres, and the inclusion of surgeons beyond their learning curve, minimising variability in surgical technique. Alternatively, this limits the generalisability of our findings to smaller, non-tertiary centres or institutions where robotic programs are in earlier stages of implementation [14]. Furthermore, as a retrospective cohort study, our analysis is subject to potential selection bias, as patient inclusion was dependent on available institutional data, potentially favouring specific patient populations. Secondly, in cases where surgeons were proficient in both robotic and laparoscopic techniques, they may have preferentially chosen one approach over the other based on specific patient characteristics. Information bias may also be present due to reliance on medical records, where documentation inconsistencies could affect data accuracy. To mitigate these risks, we implemented standardised data extraction protocols across centres. Additionally, the absence of a direct comparison with other minimally invasive techniques precludes definitive conclusions regarding the superiority of robot-assisted surgery. Therefore, future studies should perform head-to-head comparisons between minimally invasive techniques. Institutional variability in perioperative care may have also influenced our results, although standardised data collection efforts were made to ensure consistency. Future research should address international variability in robotic surgery to provide insight in potential differences in surgical techniques, patient selection, and healthcare system factors. Lastly, the lack of five-year survival data in our cohort is a limitation, and further studies with extended follow-up are required to validate the results reported in this study.

## 5. Conclusions

This multicentre, retrospective cohort study demonstrates that R-TME can be performed safely and effectively in high-volume centres by experienced surgeons, yielding promising short-term outcomes in terms of low conversion rates, acceptable complication rates, and oncological safety. However, further prospective studies with long-term follow-up are needed to substantiate these findings and clarify the role of robotic platforms in the management of rectal cancer.

## Figures and Tables

**Table 1:** Baseline characteristics of robot-assisted total mesorectal excision.

	R-TME (n = 1390)
Age (mean (SD))	67 (11)
Sex	
Male	907 (65.3)
Female	483 (34.7)
BMI (mean (SD))	26.3 (4.4)
Categorical BMI	
Underweight (BMI < 18.5)	21 (1.5)
Normal weight (BMI 18.5–24.9)	557 (40.2)
Overweight (BMI 25.0–29.9)	563 (40.6)
Obese (BMI > 30.0)	245 (17.7)
Missing	4
Comorbidities Diabetes	
No	1230 (88.9)
Yes	154 (11.1)
Missing	6
Cardiovascular	
No	912 (69.4)
Yes	403 (30.6)
Missing	75
ASA physical status score	
1	229 (16.5)
2	871 (62.7)
3	283 (20.4)
4	7 (0.5)
Previous abdominal surgery	
No	1001 (72.6)
Yes	378 (27.4)
Missing	11
Clinical T stage	
cT1	43 (3.1)
cT2	375 (27.5)
cT3	830 (60.8)
cT4	118 (8.6)
Missing	24
Clinical N stage	
cN0	532 (46.2)
cN1	420 (36.5)
cN2	199 (17.3)
Missing	239
Clinical M stage	
cM0	1231 (92.6)
cM1	98 (7.4)
Missing	61
MRF involvement on MRI	
MRF−	735 (55.9)
MRF+	580 (44.1)
Missing	75
EMVI involvement on MRI	
EMVI−	413 (45.2)
EMVI+	501 (54.8)
Missing	476
Tumour location in lumen determined on MRI	
Anterior	187 (14.6)
Circumferential	546 (42.6)
Lateral	122 (9.5)
Posterior	139 (10.8)
Semi-circumferential	13 (1)
Other	276 (21.5)
Missing	107
Tumour distance from ARJ (median [IQR])	5.00 [2.00, 7.00]
Neoadjuvant therapy	
Total (%) received NAT	830 (59.7)
Only chemotherapy	41 (4.9)
Short-course radiotherapy	225 (27.1)
Chemoradiotherapy	283 (34.1)
Total neoadjuvant therapy	281 (33.8)
ycT-stage	
ycT0	34 (7.2)
ycT1	16 (3.4)
ycT2	124 (24.5)
ycT3	256 (54.7)
ycT4	38 (8.1)
Missing	362
ycN-stage	
ycN0	306 (65.4)
ycN1	138 (29.5)
ycN2	24 (5.1)
Missing	362
MRF involvement on preoperative MRI after neoadjuvant therapy	
yMRF−	172 (54.1)
yMRF+	146 (44.9)
Missing	512
Tumour response	
No response	60 (15.7)
Partial response	261 (68.1)
Complete response	62 (16.2)
Missing	447

Abbreviations: ARJ, anorectal junction; ASA, American Society of Anesthesiology; BMI, body mass index; EMVI, extramural vascular invasion; IQR, interquartile range; MRF, mesorectal fascia; MRI, magnetic resonance imaging; n, number of patients; NAT, neoadjuvant therapy; R-TME, robot-assisted total mesorectal excision, SD, standard deviation.

**Table 2:** Intraoperative details of robot-assisted total mesorectal excision.

	R-TME (n = 1390)
Procedure type	
RLAR	842 (60.6)
NRLAR	199 (14.3)
APR	349 (25.1)
Operative time (minutes) (median [IQR])	223 [171.00, 273.50]
Intraoperative complications	
No	1148 (94.5)
Yes	67 (5.5)
Missing	175
Type of intraoperative complication	
Bleed	3 (4.5)
Perforation	20 (29.9)
Other	44 (65.7)
Conversion	
No	1338 (96.3)
Yes	52 (3.7)
Fluorescence used for evaluation of perfusion *	
No	446 (54.3)
Yes	376 (45.7)
Missing	20
Stoma *	
No	350 (41.6)
Yes	492 (58.4)
Stoma type	
Ileostomy	490 (99.6)
Colostomy	2 (0.4)

Abbreviations: APR, abdominoperineal resection; IQR, interquartile range; n, number of patients; NRLAR, non-restorative low anterior resection; R-TME, robot-assisted total mesorectal excision; RLAR, restorative low anterior resection. * This variable was calculated for patients that underwent RLAR.

**Table 3:** Postoperative outcomes of robot-assisted total mesorectal excision.

	R-TME (n = 1390)
Length of stay (days) (median [IQR])	7.00 [5.00, 11.00]
Postoperative complications within 31 days	
No	750 (71.3)
Yes	302 (28.7)
Missing	338
Ileus *	
No	1037 (89.5)
Yes	122 (10.5)
Missing	231
Wound infection *	
No	1105 (94.3)
Yes	67 (5.7)
Missing	218
Bleed *	
No	1138 (97.9)
Yes	25 (2.1)
Missing	227
Other complication *	
No	1020 (91.4)
Yes	96 (8.6)
Missing	274
Readmission within 30 days	
No	1197 (86.4)
Yes	189 (13.6)
Missing	4
Reintervention within 31 days	
No	1244 (89.6)
Yes	144 (10.4)
Missing	2
Type of reintervention within 31 days	
Radiologic	14 (10.5)
Laparoscopic surgery	42 (31.6)
Open surgery	49 (36.8)
Transanal surgery	13 (9.8)
Endoscopic intervention	2 (1.5)
Other	13 (9.8)
Missing	11
Clavien–Dindo classification *	
None	769 (55.4)
I	131 (9.4)
II	262 (18.9)
IIIa	38 (2.7)
IIIb	146 (10.5)
IV	6 (0.4)
IVa	17 (1.2)
IVb	5 (0.4)
V	14 (1.0)
Missing	2
Anastomotic leakage **	
No	667 (85.3)
Yes	115 (14.7)
Missing	60
Time	
Early (≤30 days)	118 (79.2)
Late (>30 days)	31 (20.8)
Missing	6
Grade	
Grade A, subclinical	9 (8)
Grade B, clinical, requiring radiologic or transanal drainage	40 (35.4)
Grade C, clinical, requiring re-laparotomy	64 (56.6)
Missing	42
Treatment	
Medicine, no reintervention	15 (13.3)
Radiological drainage of abscess	12 (10.6)
Transanal drainage of abscess	9 (7.9)
Endosponge	4 (3.5)
Relaparotomy with deviating stoma	23 (20.4)
Relaparotomy with reversal of anastomosis and endostomy	28 (24.8)
Other	22 (19.5)
Missing	42

Abbreviations: IQR, interquartile range; n, number of patients; R-TME, robot-assisted total mesorectal excision. * This variable accounts for postoperative complications over the entire follow up. ** This variable was calculated for patients that underwent RLAR.

**Table 4:** Pathological outcomes of robot-assisted total mesorectal excision.

	R-TME (n = 1390)
pT-staging	
pT0	104 (7.5)
pT1	123 (8.9)
pT2	468 (33.8)
pT3	632 (45.7)
pT4	29 (2.1)
pTx	27 (2.0)
Missing	7
pN-staging	
pN0	882 (63.6)
pN1	390 (28.1)
pN2	96 (6.9)
pNx	18 (1.3)
Missing	4
Quality of TME	
Complete	1105 (81.9)
Nearly complete	178 (13.1)
Incomplete	71 (5.2)
Missing	36
CRM−	1180 (94.5)
CRM+	69 (5.5)
Missing	141
Resection	
R0	1296 (94.7)
R1	69 (5.0)
R2	3 (0.2)
Missing	36
Perforation	
No	1070 (96.7)
Yes	36 (3.3)
Missing	284
Pathological nodes removed (mean (SD))	17.3 (8.94)
Positive nodes (median [IQR])	0 [0.00, 1.00]

Abbreviations: CRM, circumferential resection margin; IQR, interquartile range; n, number of patients; R-TME, robot-assisted total mesorectal excision; SD, standard deviation; TME, total mesorectal excision.

**Table 5:** Long-term oncological and survival outcomes of robot-assisted total mesorectal excision.

	R-TME (n = 1390)
Adjuvant chemotherapy	
No	1067 (82.3)
Yes	230 (17.7)
Missing	93
3-year local recurrence rate	
No	1317 (97.2)
Yes	38 (2.9)
Missing	35
Location	
Anterior Above	0
Anterior Below	4 (12.5)
Inferior	2 (6.3)
Central anastomotic	8 (25)
Central non-anastomotic	10 (31.3)
Posterior	5 (14.6)
Lateral at right	2 (6.3)
Lateral at left	1 (3.1)
Peritoneal reflection	0
Missing	6
3-year systemic recurrence rate	
No	1155 (85.4)
Yes	198 (14.6)
Missing	37
Location	
Lung	109 (56.8)
Liver	66 (34.4)
Peritoneal	4 (2.1)
Bone	1 (0.5)
Brain	1 (0.5)
Other	11 (5.7)
Missing	6
3-year overall survival	
No	136 (9.9)
Yes	1238 (90.1)
Missing	16
3-year disease free survival	
No	157 (11.4)
Yes	1215 (88.6)
Missing	18

Abbreviations: n, number of patients; R-TME, robot-assisted total mesorectal excision.

## Data Availability

The research data supporting the findings of this study are not made publicly available due to data protection regulations. Anonymized data may be made available upon reasonable request for academic purposes, subject to approval by the participating centres and relevant ethics committees.

## Supplementary Materials

The following supporting information can be downloaded at: https://www.mdpi.com/article/10.3390/cancers17081268/s1, Supplementary File S1: STROBE Checklist.

## Abbreviations

The following abbreviations are used in this manuscript:

APR	Abdominoperineal resection
ARJ	Anorectal junction
ASA	American Society of Anesthesiology
BMI	Body mass index
CRM	Circumferential resection margin
CT	Computed tomography
ERAS	Enhanced recovery after surgery
EMVI	Extramural vascular invasion
ICG	Indocyanine green
ISREC	International Study Group of Rectal Cancer
IQR	Interquartile range
L-TME	Laparoscopic total mesorectal excision
MRI	Magnetic resonance imaging
MRF	Mesorectal fascia
nCRT	Neoadjuvant chemoradiotherapy
NAT	Neoadjuvant therapy
NRLAR	Non-restorative low anterior resection
PME	Partial mesorectal excision
RCT	Randomised controlled trial
RLAR	Restorative low anterior resection
R-TME	Robot-assisted total mesorectal excision
SCRT	Short-course radiotherapy
SD	Standard deviation
STROBE	Strengthening the reporting of observational studies in epidemiology guidelines for observational studies
TNT	Total neoadjuvant therapy
TME	Total mesorectal excision
TRG	Tumour regression grade

## Appendix A

Baseline characteristics included nation (Netherlands, France, or United Kingdom), sex (male/female), age at time of surgery (years), BMI categorical ((Underweight (BMI < 18.5), Normal weight (BMI 18.5–24.9), Overweight (BMI 25.0–29.9), Obese (BMI > 30.0)), diabetic comorbidity (yes/no), cardiovascular comorbidity (yes/no), American Society of Anesthesiologists physical status (ASA) classification [31] (I, II, III, or IV), history of abdominal surgery (yes/no), preoperative T-staging (T1, T2, T3, or T4), preoperative N-staging (N0, N1, or N2), preoperative M-staging (M0 or M1), preoperative MRF-staging on MRI (negative or positive, defined as a tumour within 1 mm from the MRF), preoperative EMVI staging on MRI (negative or positive), tumour location in lumen on MRI (anterior, circumferential, lateral, posterior, semi-circumferential), tumour distance from the ARJ on MRI in cm, neoadjuvant treatment (yes/no), type of neoadjuvant treatment (none, only chemotherapy, SCRT, nCRT, chemoradiation + chemotherapy (TNT), or other), and tumour response (no response, partial response, or complete response, defined according to TRG on MRI).

Intraoperative outcomes included procedure type (RLAR, non-restorative LAR (NRLAR), or APR), operative time in minutes, intraoperative complications (yes/no), intraoperative complication type (bleeding, perforation, other), conversion (yes/no, defined as any unplanned extension of the extraction site during surgery), fluorescence used for evaluation of perfusion (yes/no), stoma placement (yes/no), and stoma type (ileostomy, colostomy).

Pathological outcomes included pathological T-staging (pT0, pT1, pT2, pT3, or pT4), pathological N-staging (pN0, pN1, or pN2), number of lymph nodes harvested, number of positive lymph nodes harvested, quality of TME (complete, nearly complete, or incomplete), completeness of resection according to Nagtegaal et al. [32] (incomplete, nearly complete, or complete), pathological CRM (negative (>1 mm) or positive (≤1 mm)), pathological resection quality (R0, R1, or R2), and tumour perforation (yes/no). Pathological staging was modified according to the American Joint Committee on Cancer 8th edition staging system during data review [33].

Postoperative outcomes included length of stay in days, postoperative surgical complications with Clavien–Dindo classification [34] (none, grade I, grade II, grade IIIa, grade IIIb, grade IV, grade IVa, grade IVb, grade V), anastomotic leakage (yes/no), ileus (yes/no), wound infection (yes/no), bleed (yes/no), other postoperative complication (yes/no), readmission within 30 days (yes/no), reintervention within 31 days (yes/no), reintervention type (radiologic, laparoscopic, open surgery, transanal surgery, endoscopic, or other), anastomotic leakage time (early (<30 days), or late (>30 days)), anastomotic leakage grade (grade A, grade B, or grade C; according to ISREC classification [26]), anastomotic leakage treatment (medicine, radiological drainage of abscess, transanal drainage of abscess, endosponge, relaparotomy with deviating stoma, relaparotomy with reversal of anastomosis and endostomy, or other), adjuvant chemotherapy (yes/no), three-year local recurrence (yes/no), location of local recurrence (anterior above, anterior below, inferior, central anastomotic, central non-anastomotic, posterior, lateral at right, lateral at left, or peritoneal reflection), three-year systemic recurrence (yes/no), location of systemic recurrence (lung, liver, peritoneal, bone, ovarian, brain, or other), three-year overall survival (yes/no), and three-year disease-free survival (yes/no). Local recurrence was identified as tumour deposits within the pelvic cavity, confirmed pathologically as adenocarcinoma or observed as growth on consecutive imaging without histopathological confirmation. Systemic recurrence was defined as distant metastasis, either pathologically confirmed or suggested by imaging showing lesion growth on successive scans.
